# Exudate and Propolis from *Escallonia pulverulenta*: Phytochemical Characterization and Antibacterial Activity

**DOI:** 10.3390/plants13141971

**Published:** 2024-07-19

**Authors:** Bairon Jorquera, Gabriela Valenzuela-Barra, Ailin Mayorga, Jessica Mejía, Gabriel Núñez, Miguel Gómez, Gloria Montenegro, Waleska E. Vera Quezada, Javier Echeverría, Adriano Costa de Camargo, Gilsane Lino von Poser, Raquel Bridi

**Affiliations:** 1 Departamento de Química Farmacológica y Toxicológica, Facultad de Ciencias Químicas y Farmacéuticas, Universidad de Chile, Santiago 8380000, Chile; bairon.jorquera@ug.uchile.cl (B.J.); gabriela.m.valenzuela@ciq.uchile.cl (G.V.-B.); ailin.mayorga@uc.cl (A.M.); 2 Departamento de Ciencias Vegetales, Facultad de Agronomía e Ingeniería Forestal, Pontificia Universidad Católica de Chile, Santiago 7820436, Chile; jcmejia@uc.cl (J.M.); ginunez@uc.cl (G.N.); mgomezu@uc.cl (M.G.); gmonten@uc.cl (G.M.); 3 Facultad de Farmacia, Escuela Química y Farmacia, Universidad de Valparaíso, Playa Ancha, Valparaíso 2340000, Chile; waleska.vera@uv.cl; 4 Centro de Investigación, Desarrollo e Innovación de Productos Bioactivos, CInBIO, Facultad de Farmacia, Universidad de Valparaíso, Valparaíso 2340000, Chile; 5 Departamento de Ciencias del Ambiente, Facultad de Química y Biología, Universidad de Santiago de Chile, Santiago 9170022, Chile; javier.echeverriam@usach.cl; 6 Nutrition and Food Technology Institute, University of Chile, Santiago 7830490, Chile; adrianoesalq@gmail.com; 7 Programa de Pós-Graduação em Ciências Farmacêuticas, Laboratório de Farmacognosia, Faculdade de Farmácia, Universidade Federal do Rio Grande do Sul, Porto Alegre 90610-000, RS, Brazil

**Keywords:** *Escallonia pulverulenta*, leaves, exudate, propolis, phenolic acids, flavonoids, antimicrobial activity

## Abstract

Propolis is a complex mixture formed from exudates that bees collect from plants and then mix with beeswax and their own salivary enzymes. Chilean propolis is characterized by the presence of phenolic compounds, which are considered responsible for the biological activities. The endemic species *Escallonia pulverulenta* (Ruiz and Pav.) Pers. [Escalloniaceae] is a recognized source of exudate to produce propolis. This study reports for the first time the chemical profile and antibacterial activity of *E. pulverulenta* exudate and leaves, as well as two samples of Chilean propolis. Palynological and morphological analysis showed the presence of *E. pulverulenta* as one of the main species in the propolis samples. UPLC-MS/MS analyses enabled the identification of phenolic acids in the leaves and in the propolis. Conversely, flavonoids are mainly present in exudates and propolis. Quercetin is the most abundant flavonol in the exudate, with similar concentrations in the propolis samples. Nevertheless, the main compound present in both samples of propolis was the flavanone pinocembrin. The antibacterial results obtained for exudate and propolis have shown a similar behavior, especially in the inhibition of *Streptococcus pyogenes*. These results show the importance of the exudates collected by the bees in the chemical composition and antibacterial capacity of propolis.

## 1. Introduction

Due to their natural origin and multiple medicinal properties, goods of bee origin have been used since the dawn of civilization in traditional medicine [1,2]. Several biological activities have been reported for propolis including anti-inflammatory, antibacterial, antifungal, antiviral, immunomodulatory properties, suppression of HIV-1 replication and immunoregulatory effect, cytotoxicity, hepatoprotection, and free radical scavenging activity [3,4,5,6]. Moreover, propolis is considered an effective substance for maintaining hygiene of the oral cavity as well as for treating caused by viral, bacterial, and fungal infections [7]. In recent years, research on natural products has focused on analyzing their chemical composition and biological properties, which vary depending on their geographical and botanical origin [8,9]. Consequently, it is estimated that bee-based products are able to inherit the properties of the plants that bees visit. This phenomenon, known as the “floral fidelity” of bees, underscores the importance of understanding the botanical origin of bee outputs such as honey, pollen, bee bread, and propolis. Bees collect nectar, pollen, and exudates from a variety of flowering plants in their foraging expeditions. These bee-derived products’ chemical compositions can reflect the diverse array of phytochemicals present in the plant sources [10].

Propolis, derived from the Greek roots “pro” (meaning “in defense of”) and “polis” (meaning “city”), is a bioactive bee product produced from exudates collected selectively from different aerial parts of plants (leaves, buds, sap flows, trichomes, and other actively exuding plant structures) [11]. These plant exudates, which may include resins, gums, and balsams, are gathered by bees and mixed with beeswax and salivary enzymes secreted by the bees themselves. Through this process, bees create a flexible and sticky resinous substance known as propolis, which they use to seal, protect, and repair structural damage to their hives from external threats (e.g., thermal insulation) and maintain humidity at constant levels [12,13]. This material is used by bees in the hive and also serves as a natural defense mechanism for bee colonies, providing antimicrobial, antifungal, and antioxidant properties that help maintain hive health. Bees strategically apply propolis to seal cracks and crevices in the hive, reinforce the hive structure, and prevent the entry of pathogens and parasites. Additionally, propolis exhibits wound-healing properties, and protects vulnerable larvae and pupae [12,14].

Within this complex mixture, phenolic compounds are particularly notable for their prominence in the composition [15,16]. Polyphenols, which include compounds such as flavonoids and phenolic acids, are abundant in propolis and contribute to its bioactivity. Flavonoids such as pinocembrin, pinobanksin, galangin and phenolic acid as caffeic acid are found in particular abundance in the propolis studied in Chile. The high concentration of phenolics in Chilean propolis samples is tied to the wide climatic diversity of the country [17,18,19,20]. Chile boasts a diverse range of ecosystems, including deserts, mountains, forests, and coastal regions, each with its unique flora. This rich botanical diversity provides bees with access to a wide variety of plant species with distinct phytochemical profiles [21].

Plants’ survival depends on their ability to develop adaptive strategies to cope with environmental adversities. One of these adaptations is to secrete or exude high and low molecular weight compounds, allowing them to create favorable microenvironments on structural surfaces [22]. Some compounds of special interest present in exudates are phenolic acids and flavonoids, since they provide great antioxidant and protective capacity against harmful compounds such as reactive oxygen species (ROS), products of constant exposure to UV radiation and pathogenic microorganisms [23,24].

In Chile, species belonging to the genus *Escallonia* (Escalloniaceae) are highly valued by honeybees (*Apis mellifera*) for producing propolis. The country boasts seven endemic species within this genus [25]. One notable species within this group is *Escallonia pulverulenta* (Ruiz and Pav.) [Escalloniaceae]. This small tree flourishes in a variety of habitats, from coastal to mountainous regions, and can be found at elevations up to 900 m above sea level. Its range extends from the Coquimbo Region in the north to the Araucanía Region in the south [26]. *Escallonia pulverulenta* has a rich history of use in traditional medicine, particularly as practiced by pre-Columbian cultures. Infusions made from this plant have been employed to treat respiratory tract ailments and as digestive stimulants. Some studies have identified a range of bioactive compounds in *Escallonia* species as kaempferol, pinocembrin, chlorogenic acid, and rutin. Iridoids such as asperuloside have also been discovered in the leaves of species within the genus [27,28].

Multiple studies have highlighted the antimicrobial properties of flavonoids found in propolis. Among these flavonoids, pinocembrin, quercetin, and apigenin have been identified as highly effective antibacterial agents. Their presence plays a crucial role in the broad-spectrum antimicrobial activity of propolis. Research shows that pinocembrin, quercetin, and apigenin can effectively inhibit various bacterial strains’ growth, thus proving to be valuable in combating microbial infections [18,19,29,30]. The antimicrobial property of propolis is often described as a synergistic effect resulting from the combination of the diverse compounds present in the resin [31].

Preliminary studies conducted with Chilean propolis of different botanical origins have demonstrated inhibitory activity against the growth of human pathogenic bacteria, including *Streptococcus pyogenes* and *Staphylococcus aureus* [19,20,30]. These findings highlight the potential therapeutic value of Chilean propolis to fight bacterial infections. The broad-spectrum antimicrobial activity exhibited by propolis underscores its importance as a natural remedy for addressing infectious diseases caused by common bacterial pathogens. The central aim of this study was to analyze the chemical profile and antibacterial activity of extracts from exudate and leaves of *Escallonia pulverulenta* as well as two samples of Chilean propolis, with the goal of establishing a match between the characteristics of the propolis and the plant species from which it originates.

## 2. Materials and Methods

### 2.1. Chemicals and Reagents

The aluminum chloride (AlCl_3_) was supplied by Merck (Darmstadt, Germany), while standards of phenolic compounds were purchased from Sigma–Aldrich (St. Louis, MO, USA). All solvents were high-performance liquid chromatography (HPLC) grade. The water was purified in a MilliQ system (Synergy, Millipore, Darmstadt, Germany).

### 2.2. Plant and Propolis Materials

*Escallonia pulverulenta* leaves were collected in the municipality of Til Til within the Metropolitan Region, Chile (1180 m, 33.08496° S, 70.92451° W), in January 2022; references are deposited at the Herbarium of the Department of Plant Sciences within the Faculty of Agronomy and Natural Systems at the Pontifical Catholic University of Chile. The plant material was dried at room temperature. The propolis samples were collected in the municipalities of Lampa (33.36757° S, 70.66426° W) and Melipilla (33.68329° S, 71.22251° W), Metropolitan Region, Chile, in 2020. The samples were stored in the dark at −20 °C until they were used. This research was conducted according to the ConPhyMP guidelines [32].

### 2.3. Botanical Analysis of Propolis Samples

For the palynological and plant structures analyses, ten grams of propolis from each of the Lampa (P1) and Melipilla (P2) samples were weighed and then mixed with 20 mL of distilled water, stirring until completely dissolved. The mixture was centrifuged at 3000 rpm for 10 min and the supernatant was discarded. The sediment was redissolved in 20 mL of distilled water and mixed by vortexing until homogeneous. A 100 µL aliquot was taken and placed on a glass slide. This was repeated a total of 4 times. A drop of red calberla was used to stain the pollen grains to allow observation under a light microscope. Subsequently, they were counted and plant structures (epidermis, trichomes, and vessels) were identified. For each slide 600 pollen grains and vegetable structures were counted. To determine botanical origin, specific literature and the botanical bee pollen catalog at the Laboratory of Botany (Department of Plant Sciences, Faculty of Agronomy and Natural Systems, Pontificia Universidad Católica de Chile, Santiago, Chile) were consulted [33]. Different structures were identified via comparison with relevant literature as well as photographs and permanent preparations available in the Laboratory of Botany (Department of Plant Sciences, Faculty of Agronomy and Natural Systems, Pontifıcia Universidad Catolica de Chile, Santiago, Chile) [34].

### 2.4. Exudate from E. pulverulenta

The exudate was obtained from previously dried leaves of *E. pulverulenta*. The leaves were weighed (11.9 g) and subsequently submerged, one by one, in methanol at room temperature. Three graduated cylinders were prepared with 50 mL of methanol, into which the sample leaves were carefully immersed with a pinch. Three consecutive immersions were performed in each graduated cylinder before moving to the next [35]. The methanol extracts were then pooled and concentrated using a rotary evaporator until dried. The resulting dry exudate extract (EE) was stored protected from light in a dryer until appropriate analyses were carried out.

### 2.5. Preparation of E. pulverulenta Leaf Extract

After obtaining the exudate, the leaves were dried at room temperature and then ground to a fine consistency. The 10 g of crushed sample was extracted with 30 mL of methanol using ultrasonic extraction (Elmasonic S10H Elma, Singem, Germany) for 15 min at room temperature according Bridi et al. [36] with minor modifications. Subsequently the solution was filtered into the vacuum, and the process was repeated three times. The pooled leaves extracts (LE) were concentrated using a rotary evaporator until dried and were then stored at −20 °C in the dark until use.

### 2.6. Preparation of Propolis Extracts

Propolis extracts (PEs) were prepared according to Bridi et al. [36] with slight modifications. A volume 30 mL of methanol p.a. were added to 3 g of previously milled raw propolis and the suspension was maintained in an ultrasonic bath (Elmasonic S10H Elma, Singem, Germany) for 30 min and then filtered. This procedure was repeated three times. The pooled propolis extracts were concentrated using a rotary evaporator until dried, and stored at −20 °C in the dark until use.

### 2.7. UPLC-MS/MS Analysis

Phenolic compounds were investigated according to described by our research group [37] with minor modifications, through an ABSciex triple Quad 4500 mass spectrometer supplied with an electrospray (TurboV) interface combined to an Eksigent Ekspert Ultra LC100 with an Ekspert Ultra LC100-XL autosampler system (AB/Sciex, Concord, ON, Canada). Electrospray in the negative mode was employed and the following parameters were used: curtain gas (CUR) = 30 psi; collision gas (CAD) = 10 psi; ion spray voltage (IS) = −4500 V; temperature (TEM) = 650 °C; ion source gas 1 (GS1) = 50 psi; ion source gas 2 (GS2) = 50 psi; entrance potential (EP) = 10 V. Chromatographic separation was carried out by employing a gradient elution with (A) 0.1% formic acid and (B) methanol as the mobile phase, using the following protocol: 0–1 min, 5% B; 1–12 min, 5–50% B; 12–13 min 50–50% B; 13–14 min, 50–5% B; and 14–15 min, 5% B. The apparatus was handled utilizing an injection volume of 10 µL, a flow rate of 0.5 mL/min, and an end-capped column (LiChrospher 100 RP-18; 125 mm × 4 mm i.d., 5 µm; Merck, Darmstadt, Germany) kept at 50 °C. Calibration curves for quantification were constructed using commercially available standards. Table 1 shows the parameters used for compound identification.

### 2.8. Antibacterial Activity

The extracts’ antibacterial activity was determined by diameter of growth inhibition against *Escherichia coli* ATCC-25922, *Staphylococcus aureus* ATCC-25923, *Salmonella typhi* ATCC-700623, *Streptococcus pyogenes* ISP 364-00, and *Listeria monocytogenes serotype* 1/2a strain BM-01-02 (1) (local isolated from cheese). The growth inhibition diameter was evaluated using the standard of CLSI (2006) [38]: bacterial strains were inoculated on Mueller Hinton agar for 24 h at 37 °C. After the incubation, colonies were selected and diluted in physiological serum to a concentration of 0.5 McFarland (1.5 × 108, Becton Dickinsson Company, Franklin Lakes, NJ, USA). Strain colonies were swabbed on the agar culture, and holes 6 mm in diameter were made. A 100 μL aliquot of the extracts (100 mg/mL; DMSO) was then deposited in each hole. Cultures were incubated between 18 and 24 h at 35 °C. Each inhibition growth diameter was measured and compared with streptomycin (1 mg/mL) as antibiotic control.

The minimum inhibitory concentration against *S. pyogenes*, *S. aureus* and *S. typhi* was calculated using a standard microdilution technique. Bacterial concentrations were determined by using 0.5 McFarland diluted in 5 mL of physiological serum; from this solution 10 μL was extracted and diluted in 10 mL of physiological serum. This allowed a concentration of approximately 10^3^ UFC. DMSO and streptomycin (1 µg/mL) was used for control. MIC values were taken as the lowest concentration that produced no visible bacterial growth (no turbidity) when compared with controls after 24 h of incubation at 37 °C.

## 3. Results and Discussion

### 3.1. Botanical Origin

The samples of propolis were analyzed employing light microscopy to determine the microstructures (trichomes, epidermal cells, and any other plant fragments) present, which are consistent evidence of their botanical origin. In addition, the study of pollen grains, which comprise around 5% of the composition of propolis, provides an indication of the geographical origin of the samples [39,40]. The results regarding the botanical origin of the propolis samples investigated in this study appear in Table 2.

In the fraction of plant debris in samples P1 and P2, pollen and structures from native and endemic species were observed, with a particular emphasis on *Quillaja saponaria*, *Lomatias hirsuta*, *Schinus latifolius*, and *Escallonia pulverulenta.* Structures commonly found in introduced species in the central region of Chile, particularly those of the genus *Populus*, were also observed in all samples. Table 2 indicates that in samples P1 and P2, *E. pulverulenta* pollen granules constituted approximately 10 and 15% of the total pollen composition, respectively. On the other hand, the structural elements accounted for 42 and 32% of the *E. pulverulenta* trichomes. Given trichomes’ typical morphology, the fragments of *E. pulverulenta* leaves can be easily recognized in samples and are typically employed for their identification. These results indicate a prevalence of *E. pulverulenta* species in the two propolis samples selected for the study.

### 3.2. Extracts Characterization

Extraction yields (% *w*/*w*) were as follows: LE (42%); EE (62%); PE1 (64%); PE2 (71%). These extracts were submitted to chemical evaluation as described in Section 2 (Materials and Methods).

#### UPLC-MS/MS

Identification and quantification of the phenolic compounds in the samples was carried out via UPLC-MS/MS. The different parameters of analysis allowed the identification of phenolic acids and flavonoids, in the exudate (EE), and leaf extract (LE) of *E. pulverulenta* as well as in propolis (EP1) and (EP2). Isoflavonoids were identified in both propolis samples (Table 3).

Secretory structures, like trichomes, can synthesize complex mixtures of various compounds, and the secretion (exudate) is chemically heterogeneous. Secretory trichomes producing phenolic compounds are reported in families such as Asteraceae, Cucurbitaceae, Lamiaceae, Oleaceae, Orobanchaceae, Verbenaceae, and others. The main classes of phenolic compounds found in the secreted material are phenolic acids, tannins, and flavonoids [41,42]. Flavonoids accumulate in the epidermal and subepidermal layers of the vegetative organs to protect plants against damage by ultraviolet radiation (280–320 nm). However, additional roles include functioning as antioxidants and allelopathic agents (phytoalexins) which further protect plants against pathogens [41,43]. The resinous shrub *E. pulverulenta* is widely distributed in the semiarid zone of Chile, known as “Norte Chico”, and the central zone, which is characterized by warm, dry summers and short, humid winters [25]. The results showed a higher concentration in the exudate than in the leaves, since the exudate was used as a defense mechanism against extreme environmental conditions [25].

The phenolic acids observed in the samples were *p*-coumaric acid, gallic acid, ferulic acid, chlorogenic acid, cryptochlorogenic acid, and caffeic acid. The presence of phenolic acids was primarily detected in the leaves and in the propolis extracts. The concentration of caffeic acid in the propolis stands out, possibly due to the contribution of exudates and resins from the other plant species present. Phytochemical studies showed that exudates are constituted by a mixture of different compounds, including flavonoids [35,42,43,44,45]. Aglycones such as apigenin and quercetin are typically accumulated on the leaf surface and secreted by glandular trichomes, a process mainly influenced by climatic and soil conditions [45]. Quercetin is the most abundant flavonol present in the exudate of *E. pulverullenta* and with similar concentrations in the propolis samples, while its glycoside rutin is found mainly in the leaf extract. Pinocembrin has been identified in various sorts of Chilean propolis [18,46]. This flavanone has not yet been described for *E. pulverullenta*, but was determined in *Escallonia illinita* C. Presl. [Escalloniaceae] [47]. In the present HPLC analysis, pinocembrin appears in the exudate and leaf extract only in trace amounts, below the limit of quantification. Nevertheless, it was the main compound in both propolis samples and it has been detected as a primary component of poplar-type propolis, as reported by several authors. Samples EP1 and EP2 have respective structural elements accounting for 16 and 22% of poplar (*Populus* sp.) in their botanical composition, along with 10 and 16% pollen content according to palynological analysis. This species is the second contributor to the elaboration of the studied propolis (Table 2), potentially justifying the high concentrations of pinocembrin (approximately 500 mg/100 g of sample). In addition, the phenolic compounds *p*-coumaric acid, ferulic acid, caffeic acid, chlorogenic acid, and apigenin have already been described in *Populus nigra* L. [Salicaceae] exudates [48]. The presence of apigenin is consistent with previous reports of apigenin derivatives such as apigenin-7,4-dimethylether, acacetin (apigenin 4′-methyl ether) and genkwanin (7-O-methylapigenin) in *E. pulvurulenta* exudates [49].

Isoflavones have been previously documented in propolis from various countries [50]. EP1 and EP2 presented daidzein traces, biochanin A 13.74 and 26.18 mg/100 g, formononetin 152.14 and 144.95 mg/100 g. In Chilean propolis, the presence of genistein and daidzein has been reported in the study conducted by Veloz et al. [51], in which the samples mainly contained of structures from *Lotus uliginosus* Schk. [Fabaceae] (58–61%). Isoflavones are a category generated almost exclusively by members of the Fabaceae plant family. In our study, the presence of two species of the Fabaceae family, *Lotus pedunculatus* and *Melilotus indicus*, could be the source of these compounds in the evaluated propolis.

### 3.3. Antibacterial Activity

In this study, we evaluated antibacterial activity using an agar diffusion test of samples against the human pathogenic microorganisms including *Streptococcus pyogenes*, *Staphylococcus aureus*, *Escherichia coli*, *Salmonella typhi*, and *Listeria monocytogenes*. Table 4 displays the mean diameters of growth inhibition zones; measured in millimeters, obtained for each tested strain. The extracts were found to be most sensitive to *S. aureus* and *S. pyogenes*, exhibiting the highest inhibition zones (24 ± 0.5 mm). Propolis effectively inhibited the growth of *S. typhi* and for all *L. monocytogenes* strains. However, no inhibitory effect was observed against *E. coli*, which was the most resistant strain.

The Minimum Inhibitory Concentrations (MICs) of the test samples are presented in Table 5. MIC was determined as the lowest concentration of the extracts that inhibited growth in the tested microorganisms. The antibacterial efficacy of honeybee products, particularly propolis, is well-documented in the literature, with Gram-positive bacteria being especially affected [14,20]. It is speculated that the effect of propolis on Gram-positive bacteria may be attributed to their lack of an outer membrane, rendering them more susceptible to damage caused by active compounds, mainly polyphenols [52]. Notably, among this group of bacteria are *S. pyogenes*, *S. aureus*, and *L. monocytogenes*. The exudate (EE) and propolis samples (EP1 and EP2) exhibited a remarkable bactericidal effect against *S. pyogenes* with MIC values less than 3 µg/mL extract. Similar values have been reported for propolis from Ireland, Czechia, and Germany [53], although this represents the first time for the exudate of *Escalonia*. The antibacterial results obtained for exudate and propolis have shown a similar behavior, especially in the inhibition of *S. pyogenes*, which can be attributed to the profile of phenolic compounds inherited by propolis from the main plant species. Some studies have proposed that structural damage to microorganisms is a possible mechanism through which propolis exerts its antimicrobial activity. However, the precise mechanism of action of propolis remain unclear, owing to the synergistic interaction of its components, and this natural substance demonstrates multi-target activity within the cell. Current applications of propolis include formulations for cold syndrome (upper respiratory tract infections, common cold, and flu-like infections), as well as dermatological preparations. *Streptococcus pyogenes* infections are a major cause of morbidity and mortality worldwide [54]. This bacteria can cause infections in the skin, soft tissue, and respiratory tract, and is a leading cause of pharyngitis in children and adolescents [55]. Our results corroborate others studies showing that propolis is very promising to treat bacterial infections of the oral cavity.

## 4. Conclusions

This research is the first study regarding the chemical composition and antimicrobial capacity of *E. pulverulenta* leaves, exudate, and propolis. The results show a great concentration of phenolic acids and flavonoids in the analyzed samples, all putatively identified using UPLC-MS/MS. Quercetin is the most abundant flavonol aglycone in the exudate, with similar concentrations in the propolis samples. The high concentration of pinocembrin in the propolis could be justified by the presence of poplar (*Populus* sp.) in the botanical composition, since it is not present in the *E. pulverulenta* exudate. Propolis presented isoflavones, mainly biochanin and formononetin. Exudate and propolis have demonstrated comparable antibacterial activity, particularly in terms of inhibiting *Streptococcus pyogenes*. These findings highlight the significance of the exudate plants collected by bees for the chemical composition and antimicrobial properties of propolis.

## Figures and Tables

**Table 1:** Parameters used for the LC–MS/MS analysis of the phenolics examined.

Compound	MRM Transition 1	DP	CE	CXP	MRM Transition 2	DP	CE	CXP
*p*-Coumaric acid	162.9 > 119.0	−70	−20	−5	162.9 > 192.8	−70	−38	−25
Gallic acid	168.9 > 124.9	−70	−18	−15	168.9 > 78.9	−70	−28	−15
Chlorogenic acid	353.1 > 191.0	−75	−22	−5	353.1 > 85.0	−75	−54	−9
Cryptochlorogenic acid	353.0 > 150.0	−60	−24	−5	353.0 > 178.9	−60	−24	−3
Ferulic acid	193.0 > 134.0	−55	−20	−7	193.0 > 177.9	−55	−16	−15
Caffeic acid	178.9 > 135.0	−70	−20	−5	178.9 > 133.9	−70	−32	−7
Catechin	289.0 > 245.0	−100	−22	−13	289.0 > 108.9	−100	−30	−7
Epicatechin	289.0 > 244.9	−110	−20	−19	289.0 > 109.0	−110	−30	−7
Formononetin	267.1 > 251.6	−110	−26	−9	267.1 > 222.9	−110	−46	−9
Apigenin	268.9 > 117.0	−130	−40	−9	268.9 > 150.9	−130	−32	−5
Biochanin A	282.9 > 267.9	−80	−32	−5	282.9 > 211.1	−80	−46	−5
Luteolin	285.0 > 133.0	−125	−42	−5	285.0 > 150.9	−125	−34	−11
Kaempferol	285.0 > 184.9	−135	−36	−15	285.0 > 116.9	−135	−48	−3
Quercetin	301.0 > 150.9	−15	−28	−13	301.0 > 178.8	−15	−24	−11
Isorhamnetin	315.0 > 299.9	−130	−32	−15	315.0 > 150.9	−130	−40	−11
Rutin	609.0 > 299.8	−170	−50	−13	609.0 > 300.5	−170	−42	−9
Myricetin	316.9 > 150.9	−105	−30	−7	316.9 > 178.9	−105	−26	−9
Pinocembrin	255.1 > 212.9	−95	−28	−7	255.1 > 151.0	−95	−28	−7

CE, [collision energy]; CXP, [collision cell exit potential]; DP, [declustering potential]; MRM, [multiple reaction monitoring].

**Table 2:** Palynological and structural-element-based identification.

Species	Pollen ^1^ (P1)	Elements ^2^ (P1)	Pollen ^1^ (P2)	Elements ^2^ (P2)
*Quillaja saponaria* Molina [Quillajaceae]	7 (6.9%)	-	-	-
*Brassica rapa* L. [Brassicaceae]	7 (6.9%)	-	-	9 (18%)
*Eucalyptus* sp. [Myrtaceae]	8 (7.8%)	6 (12%)	-	-
*Melilotus indicus* (L.) All. [Fabaceae]	9 (8.8%)	-	-	-
*Escallonia pulverulenta* (Ruiz and Pav.) Pers. [Escalloniaceae]	10 (9.8%)	21 (42%)	15 (14.9%)	16 (32%)
*Populus* sp. [Salicaceae]	10 (9.8%)	8 (16%)	16 (15.8%)	11 (22%)
*Schinus latifolia* (Gillies ex Lindl.) Engl. [Anacardiaceae]	12 (11.7%)	-	-	-
*Carduus* sp. [Asteraceae]	13 (12.7%)	6 (12%)	-	-
*Lotus pedunculatus Cav.* [Fabaceae]	26 (25.6%)	-	15 (14.9%)	-
*Lomatia hirsuta* (Lam.) Diels [Proteaceae]	-	-	9 (8.7%)	-
*Raphanus sativus* L. [Brassicaceae]	-	-	12 (11.9%)	-
*Rubus ulmifolius* Schott [Rosaceae]	-	-	34 (33.8%)	10 (10%)
*Retanilla trinervia* (Gillies and Hook.) Hook and Arn. [Rhamnaceae]	-	9 (18%)	-	4 (8%)
TOTAL	102 (100%)	50 (100%)	101 (100%)	50 (100%)

^1^ Number of pollen granules counted in one field. ^2^ Number of structural elements (trichomes, vessels, epidermis) counted in one field.

**Table 3:** Quantification of phenolic compounds by UPLC-MS/MS for leaf extract (LE), exudate (EE), propolis (EP1) and (EP2) samples of *E. pulverulenta*. Values correspond to the average data with their respective S.D. in mg/100 g of sample (*n* = 3).

	Quantificaction of Phenolic Compounds (mg/100 g)			
	LE	EE	EP1	EP2
Phenolic acids				
*p*-Coumaric acid	3.95 ± 0.21		28.63 ± 0.04	28.65 ± 0.18
Gallic acid	tr	nd	nd	1.60 ± 0.19
Ferulic acid	tr	nd	13.63 ± 0.28	12.68 ± 0.59
Chlorogenic acid	40.64 ± 1.99	tr	7.69 ± 0.21	nd
Cryptochlorogenic acid *	11.53 ± 0.98	nd	1.22 ± 0.06	Tr
Caffeic acid	2.91 ± 0.14	tr	48.43 ± 2.34	43.80 ± 2.03
Flavonoids				
Catechin	60.84 ± 0.25	nd	nd	nd
Epicatechin	93.97 ± 0.94	nd	nd	nd
Isorhamnetin	11.43 ± 1.77	nd	nd	nd
Pinocembrin	tr	tr	528.23 ± 1.34	587.39 ± 4.37
Luteolin	0.97 ± 0.33	47.03 ± 1.82	10.23 ± 0.11	11.17 ± 3.28
Kaempferol	tr	1.95 ± 0.74	35.76 ± 0.79	26.66 ± 4.66
Apigenin	tr	75.78 ± 4.99	43.03 ± 3.85	35.00 ± 5.28
Myricetin	1.54 ± 0.05	nd	1.41 ± 0.01	1.04 ± 0.01
Rutin	175.88 ± 10.98	1.06 ± 0.10	4.99 ± 0.03	1.70 ± 0.32
Quercetin	11.25 ± 2.21	163.08 ± 6.01	142.23 ± 3.77	145.35 ± 7.02
Isoflavonoids				
Daidzein	nd	nd	tr	tr
Formononetin	nd	nd	152.14 ± 2.12	144.95 ± 6.84
Biochanin A	nd	nd	13.74 ± 1.49	26.18 ± 0.00

tr, trace; nd, not detected. * Cryptochlorogenic acid was quantified as equivalents of gallic acid. Results expressed as mean ± standard deviation of three replicates.

**Table 4:** Inhibition zone diameter (mm) in agar diffusion tests. The values represent the average distance of inhibition zone diameters for the different samples (*n* = 3).

	Inhibition Zone Diameter (mm)					
Bacterial Type Strains	LE	EE	EP1	EP2	DMSO	Streptomycin
*Streptococcus pyogenes*	-	16	24	24	-	25
*Staphylococcus aureus*	17	17	24	24	-	28
*Salmonella typhi*		-	10	12	-	29
*Escherichia coli*		-	-	-	-	32
*Listeria monocytogenes 1/2a*	-	-	17	17	-	25

(-) Absence of inhibition zone.

**Table 5:** MIC required to start inhibiting bacterial strain growth; values correspond to the average data in mg/mL (n = 3).

	Minimum Inhibitory Concentrations (mg/mL)			
Bacterial Type Strains	LE	EE	EP1	EP2
*Streptococcus pyogenes*	3.125	<0.003	<0.003	<0.003
*Staphylococcus aureus*	6.250	12.50	12.50	12.50
*Salmonella typhi*	100.0	100.0	100.0	50.00
*Listeria monocytogenes 1/2a*	3.125	50.00	1.562	1.562

## Data Availability

The data presented in this study are available on request from the R.B.
